# Factors Influencing Habitual Physical Activity in Parkinson’s Disease: Considering the Psychosocial State and Wellbeing of People with Parkinson’s and Their Carers

**DOI:** 10.3390/s22030871

**Published:** 2022-01-24

**Authors:** Ríona Mc Ardle, Silvia Del Din, Rosie Morris, Lisa Alcock, Alison J. Yarnall, David J. Burn, Lynn Rochester, Rachael A. Lawson, on behalf of the ICICLE-PD Study Group

**Affiliations:** 1 Faculty of Medical Sciences, Translational and Clinical Research Institute, Newcastle University, Newcastle Upon Tyne NE4 5TG, UK; riona.mcardle@ncl.ac.uk (R.M.A.); silvia.del-din@ncl.ac.uk (S.D.D.); lisa.alcock@ncl.ac.uk (L.A.); alison.yarnall@ncl.ac.uk (A.J.Y.); lynn.rochester@ncl.ac.uk (L.R.); 2 Sport, Exercise and Rehabilitation Department, Northumbria University, Newcastle Upon Tyne NE7 7YT, UK; ROSIE.E.MORRIS@NORTHUMBRIA.AC.UK; 3 Newcastle Upon Tyne Hospital NHS Foundation Trust, Newcastle Upon Tyne NE7 7DN, UK; david.burn@ncl.ac.uk; 4 Faculty of Medical Sciences, Population Health Institute, Newcastle University, Newcastle Upon Tyne NE7 7YT, UK

**Keywords:** Parkinson’s disease, habitual physical activity, wearable technology, wellbeing, carer, accelerometer, psychosocial, remote monitoring

## Abstract

Participating in habitual physical activity (HPA) may slow onset of dependency and disability for people with Parkinson’s disease (PwP). While cognitive and physical determinants of HPA are well understood, psychosocial influences are not. This pilot study aimed to identify psychosocial factors associated with HPA to guide future intervention development. Sixty-four PwP participated in this study; forty had carer informants. PwP participants wore a tri-axial accelerometer on the lower back continuously for seven days at two timepoints (18 months apart), measuring volume, pattern and variability of HPA. Linear mixed effects analysis identified relationships between demographic, clinical and psychosocial data and HPA from baseline to 18 months. Key results in PwP with carers indicated that carer anxiety and depression were associated with increased HPA volume (*p* < 0.01), while poorer carer self-care was associated with reduced volume of HPA over 18 months (*p* < 0.01). Greater carer strain was associated with taking longer walking bouts after 18 months (*p* < 0.01). Greater carer depression was associated with lower variability of HPA cross-sectionally (*p* = 0.009). This pilot study provides preliminary novel evidence that psychosocial outcomes from PwP’s carers may impact HPA in Parkinson’s disease. Interventions to improve HPA could target both PwP and carers and consider approaches that also support psychosocial wellbeing.

## 1. Introduction

Parkinson’s disease (PD; see Supplementary Table S1 for table of abbreviations) is a progressive neurodegenerative disorder, accompanied by loss of independence and function over the disease course [1]. As this has negative consequences for individual wellbeing [2], strategies to decelerate progression to dependence and disability are important to maintain quality of life (QoL) in people with Parkinson’s disease (PwP).

Supporting PwP to participate in habitual physical activity (HPA) may be an important mediating factor for QoL and functional abilities [3]. HPA refers to any movement which is produced by skeletal muscles and requires energy expenditure [4]. This does not have to be structured exercise, and encompasses movements people participate in every day, such as walking around their home, doing their gardening, or going out into their communities. PwP engage in significantly less HPA compared to normal ageing, spending less time walking and taking fewer steps per day [5,6,7], with volume of activity significantly decreasing annually [8]. They demonstrate different patterns and variability of activity, engaging in shorter, less variable walking bouts compared to older adult controls [5]. Additionally, lower levels of HPA are associated with worse cognitive impairments, lower mood and wellbeing, greater falls risk, dependence and higher perceived disability [7,9]. Studies in older adults indicate that increasing HPA relative to their baseline volume may be beneficial for functional abilities [10]. Therefore, interventions to increase or maintain HPA may be an inexpensive and inclusive strategy to slow down loss of independence and functional abilities following PD diagnosis.

In order to effectively develop interventions relating to HPA, we need to first identify modifiable predictors of HPA in PD. There is a lack of research investigating psychosocial influences of HPA, such as emotional wellbeing and support from carers. As both PwP and carers have worse psychosocial outcomes compared to the general population [11], and associations between PD and carer wellbeing has been previously shown [12], psychosocial determinants may be key targets for prospective interventions to preserve HPA, maintaining functional abilities and QoL.

Therefore, this pilot study aimed to: (1) examine HPA over time in PwP, with and without carer support, (2) identify cognitive, physical and psychosocial factors associated with HPA, and (3) determine predictors of change in HPA in overall PD and in PwP with carers only. We hypothesised that (1) PwP will significantly decrease HPA over time, (2) poorer cognitive, physical and psychosocial function in PwP will be associated with lower levels and greater decline in HPA over time, and (3) poorer psychosocial function in carers will also be associated with lower levels and decline in HPA over time.

## 2. Materials and Methods

### 2.1. Participants

This study was part of the Incidence of Cognitive Impairment in Cohorts of Longitudinal Evaluation–Parkinson’s disease (ICICLE-PD) study [13]. Newly-diagnosed PwP were recruited from outpatient clinics and the community in Newcastle and Gateshead, UK, between June 2009 and December 2011. A movement disorder specialist diagnosed idiopathic PD according to the Queen’s Square Brain Bank Criteria [1]. Full inclusion and exclusion criteria have been previously reported [13]. In brief, participants were excluded at baseline if they demonstrated significant cognitive impairment (Mini Mental State Examination <24) or dementia, as characterised by Movement Disorder Society criteria [14], if they had dementia with Lewy bodies, drug-induced or vascular Parkinsonism, atypical Parkinson’s syndromes, poor English language or the inability to consent. It should be noted that there were no inclusion criteria based on disease severity, such as Hoehn and Yahr class. All participants were assessed in an “on” motor state; levodopa equivalent daily dose (LEDD) was calculated [15]. All participants provided informed written consent. The Newcastle and North Tyneside Research Ethics committee approved the study.

Participants were subsequently reassessed at 18 months intervals over 54 months. This analysis includes PwP who were assessed for HPA at 36 months (referred to as baseline in this manuscript) and 54 months (referred to as follow-up) from entering the study. This was to include carer assessments, which were included at a 36-month follow-up [12]. Informal carers were partners, spouses, adult family members or friends of the participant with Parkinson’s, who were the primary caregiver of PwP and also provided written informed consent.

### 2.2. Measurement of Habitual Physical Activity

At both time points, a body-worn monitor (Axivity AX3, York, UK; dimensions 23.0 × 32.5 × 7.6 mm; weight: 11 g, sampling frequency 100 Hz, range ± 8 g, memory: 512 Mb, Battery life: up to 30 days at 12.4 Hz, up to 14 days at 100 Hz) was placed on the participants’ lower back, on the fifth lumbar vertebra. The monitor was fixed directly to the skin with gel adhesive (PALStickies, PAL Technologies, Glasgow, UK) and a hypo-allergenic plaster (Hypafix BSN Medical Limited, Hull, UK). They were asked to wear it continuously for seven days. The monitor is shower-proof and can be worn to bed, and participants were provided instructions and additional materials should the need to reattach it occur. Data were downloaded to a computer and segmented by day. A MATLAB programme was used to carry out analysis; it is described in brief elsewhere [16,17,18]. 

Accelerometer signals were transformed to a horizontal vertical co-ordinate system. To identify walking bouts (continuous periods of walking), raw acceleration data were filtered using a second-order low-pass Butterworth two-pass digital filter, with a cut-off frequency of 17 Hz [16]. Walking bouts (i.e., any continuous period of walking) were identified for each day by applying selective thresholds on the vector magnitude and standard deviations of tri-axial acceleration signals [15,17]. All walking bouts greater than 3 steps were included for data outcomes, with no resting threshold applied. Once walking bouts were identified raw acceleration signals were filtered with a low-pass, fourth-order Butterworth filter with cut-off frequency of 20 Hz [19]. A Gaussian continuous wavelet transform of vertical acceleration was then applied to identify initial contacts and final contacts, which allowed identification of steps. For each bout, total steps per bout and bout length were calculated, and the total number of bouts was calculated through bout identification. The step detection and gait outcome quantification algorithm has been thoroughly validated in laboratory conditions against a gold standard (GAITRite instrumented walkway; a pressure sensitive walkway) [19] and results show excellent agreement for mean gait characteristics, including step velocity (ICC(2,1) = 0.928). Step count agreement was also excellent (r > 0.900) for the same algorithm [20]. Outcomes derived from the body-worn monitors included characteristics of volume, pattern, and variability of HPA. Volume includes total walk time, total steps, and total walking bouts per day. Pattern characteristics include mean length of walking bouts and alpha scores. Alpha describes the distribution of walking bouts, representing the ratio of short to long bouts scaled relative to the individual’s shortest walking bout [17,18]. Higher alpha scores indicate that individuals spend proportionately more time in short walking bouts compared to long ones. Variability (S2) of HPA is characterised by variability of walking bout length between different bouts and describes how widespread the data is, illustrating how much bout length changes across the time period. 

### 2.3. Clinical, Cognitive and Psychosocial Outcomes for PD

Demographic information for PwP were collected, including age, sex, and years of education. We used a range of validated questionnaires to determine psychosocial outcomes in caregivers. At baseline, motor disease severity was measured using the MDS Unified Parkinson’s Disease Rating Scale Part III (MDS-UPDRS III); participants were assessed in an “on” motor state. Levodopa equivalent dose was calculated for all dopaminergic medications. The Geriatric Depression Scale (GDS-15) assessed depression [21]. Quality of life was assessed using the Parkinson’s Disease Questionnaire (PDQ-39) summary index (SI) and eight sub-scales: mobility, emotional wellbeing, activities of daily living, communication, cognition, stigma, support and body discomfort [22]; scores ranged from 0 (best possible QoL) to 100 (worst possible QoL). Global cognitive function was assessed using the Montreal Cognitive Assessment (MoCA). The FAS verbal fluency test measured executive function in PD [23]. Gait speed represented a measure of physical function and was measured by participants completing four intermittent walks across a GaitRite instrumented walkway (7 m long × 0.6 m wide), starting and ending 1.5 m from the walkway [24]. 

### 2.4. Clinical and Psychosocial Outcomes for Carers

Demographic information for carers was collected, including age, sex, education, and weekly hours spent caregiving. We used a range of validated questionnaires to determine psychosocial outcomes in caregivers. At baseline, anxiety and depression were measured using the Hospital Anxiety and Depression Scale (HADS) with subscales for anxiety (HADS-A) and depression (HADS-D) [25]. Quality of life for carers was measured with the Parkinson’s Disease Questionnaire for Carers (PDQ-Carer) [26]. The PDQ-carer comprises a SI and four subscales: social and personal activities, anxiety and depression, self-care, and stress; scores ranged from 0 (best possible QoL) to 100 (worst possible QoL).

### 2.5. Data Analysis

Statistical analysis was conducted using SPSS (IBM Corp. V.24, Armonk, NY, USA). Normality of data was assessed by inspection of boxplots, histograms, and Shapiro–Wilk tests. Independent *t*-tests, Mann–Whitney U tests and Chi-squared tests, as appropriate, identified differences between PwP with and without carers. 

R software (Version 4.0.4; R Foundation for Statistical Computing, Vienna, Austria) and *lme4* were used to perform linear mixed effects analysis of the relationship between baseline demographic, clinical and psychosocial data, and HPA from baseline to 18 months. A random intercept model was used, where the intercept varied at the participant and time level. For all HPA measures including all PD participants, PD age, sex, motor severity (MDS-UPDRS III), global cognition (MoCA), executive function score (FAS), carer status and quality of life total and sub-scores (PDQ-39 SI and subscales for mobility, emotional wellbeing, activities of daily living, communication, cognition, stigma, support, and body discomfort) as fixed effects, as well as interactions with time (variable × Time) for each measure. A reduced model was produced by excluding non-significant predictors to which cognitive measures were added. Fit of the models was assessed by likelihood ratio tests. Non-significant predictors were retained if excluding them significantly reduced the fit of the model. This analysis was repeated in PwP who had carers only to determine associations with carer psychosocial measures. In addition to the measures listed above, carer age, hours caring per week, carer anxiety and depression (HADS-A and HADS-D, respectively) and carer quality of life total and subscales (PDQ-Carer SI and social and personal activities, anxiety and depression, self-care, and stress) were entered into the model as fixed effects as well as interactions with time for all variables. For all analyses, we applied Benjamini–Hochberg multiple comparisons correction with a 5% false discovery rate.

## 3. Results

### 3.1. Participants

HPA was assessed in 64 PwP assessed at baseline. Of these participants, 40 (62.5%) had informal carers (Table 1). At the 36-month follow-up (i.e., baseline for this analysis), the mean time since PD diagnosis was 44.3 ± 4.3 months. PwP with carers were older (mean 71.5 ± 8.7 vs. 64.5 ± 10.7, respectively, *p* = 0.006), more likely to be male (25% female in PwP with carers vs. 54.2% female in PwP without carers, respectively, *p* = 0.0.19) and demonstrated higher scores on the PDQ Cognition subscale (median score: 25.8 vs. 18.0, respectively, *p* = 0.016) compared to PwP without carers. There were no other significant differences between groups (*p* > 0.05 for all).

Forty carers participated in the pilot study (Supplementary Table S2). Their mean age was 68.3 ± 10.0 years and 77.6% were female. The majority of carers (n = 38, 95%) were the spouse or partner of a PwP, 2.5% were a relative and 2.5% were a friend of the participant. 95% lived with the PwP and 65% were retired from work. On average, carers had spent 24 ± 26 months caring for their care recipient, accounting for a mean of 40 ± 63 h per week. Mean scores for anxiety (HADS anxiety: 4 ± 4) and depression (HADS depression: 3 ± 3) were considered normal. 

### 3.2. Does Habitual Physical Activity Decline over Time in People with PD?

There were no significant between-group differences for HPA found at baseline or follow up between PwP with and without carers (Table 2). In the overall group (n = 64), there were no significant main effects for time, indicating that HPA does not significantly decline over time. Considering all measures of HPA, in PwP with carers, mean bout duration was the only outcome that significantly increased over time (β = 21.7, *p* < 0.001).

### 3.3. Which Baseline Factors Influence Habitual Physical Activity in People with PD?

Linear mixed effects modelling determined baseline associations with change in HPA measures between baseline and follow-up in the overall group (n = 64; Table 3). 

Having a carer was not significantly associated with differences in any HPA measure or in change over time (*p* > 0.05 for all). Greater executive function (FAS), and faster step velocity were associated with greater time spent walking per day, greater steps and bouts per day, cross-sectionally (*p* < 0.01 for all). Worse PDQ-39 cognition scores were associated with more time spent walking (*p* = 0.0006). Greater motor disease severity and poorer executive function were associated with an increase in mean bout length duration per day over time (*p* < 0.01 for both). Cross-sectionally, higher variability was associated with faster step velocity (*p* = 0.001). Older participants had a higher alpha score (*p* < 0.001) and increased alpha over time was associated with poorer perceived social support (PDQ-39 social support, *p* = 0.005) at baseline.

### 3.4. Which Factors Influence Habitual Activity in People with PD with Carers at Baseline?

Linear mixed effects modelling determined baseline associations with change in HPA measures between baseline and follow-up in participants with carers only (Table 4).

Greater time spent walking per day in PwP was associated with faster baseline step velocity (*p* < 0.001) and lower carer anxiety (HADS-A, *p* = 0.008) when evaluated cross-sectionally. Similarly, more steps per day in PwP was associated with faster step velocity (*p* < 0.001). Poorer carer perceived anxiety and depression were associated with increased steps over time in PwP, but poorer carer self-care was associated with reduced steps over time (*p* < 0.05 for all).

Higher step velocity and greater executive function (FAS scores) were associated with a greater number of bouts per day cross-sectionally (*p* < 0.01 for all). However, increased perceived carer anxiety and depression was associated with increased number of bouts per day in PwP (*p* = 0.007), while poorer carer self-care was associated with a decline in bouts per day in PwP (*p* = 0.002). Increased mean bout duration in PwP was associated with more hours caring per week at baseline (*p* < 0.001). Increased bout duration over time was associated with faster step velocity, greater global cognition and executive function and greater carer strain at baseline (*p* < 0.01 for all), while decline in bout duration over time in PwP was associated with carers spending more hours caring per week at baseline (*p* < 0.001).

Greater baseline carer depression (*p* = 0.009) was associated with lower variability in HPA, while greater perceived strain in carers (*p* = 0.005) was associated with higher variability in PwP cross-sectionally. Poorer perceived social support in PwP (PDQ-39 social support) was associated with an increase in alpha scores over time (*p* = 0.010).

## 4. Discussion

The key aims of this pilot study were to examine change in HPA over 18 months in PwP, and to identify significant cognitive, physical, and psychosocial influences on HPA in PwP overall (irrespective of carer or no carer), and for PwP with carers only. Key findings suggest that HPA participation in PwP is somewhat explained by cognitive and physical function, limited social support and worse carer psychosocial outcomes (i.e., anxiety and self-care). These findings suggest that supporting PwP’s cognitive, physical, and psychosocial wellbeing and the psychosocial wellbeing of their carers, may help them to maintain their HPA following PD diagnosis. This support could complement and extend the traditional exercise model by providing adaptive coping strategies for both PwP and their carers. 

### 4.1. Does Habitual Physical Activity Decline over Time in People with PD?

Our results indicated that no HPA characteristics significantly declined or changed over 18 months, contradicting previous literature and hypothesis 1 [8]. However, participants in this pilot study were three years post-diagnosis; their HPA may have changed prior to this timepoint due to other factors, such as reduced social networks or lost interest in hobbies. Compared to previously reported results for normal ageing [17], HPA appears lower in volume, less variable with different patterns (shorter bout length and higher alpha) in this PD group, potentially supporting this explanation. It should also be noted that volume of HPA shows trends of decline (e.g., from an average of 161 to 137 min walking per day; see Table 2), but perhaps our sample size was too small to capture this change. 

### 4.2. Which Individual Characteristics Influence Habitual Physical Activity?

Consistent with the literature, greater cognitive impairment (i.e., executive function) and worse physical function (i.e., slower gait velocity) explained and predicted lower volume of HPA in PwP [7]. These findings highlight the important interplay of cognition and motor function to maintain our daily movement and functional abilities. This association has been previously found in both the quantity (i.e., habitual physical activity) and quality of gait (e.g., spatio-temporal characteristics) in PD. For example, Loprinzi, et al. [27] reported a favourable association between greater moderate-vigorous physical activity and global cognitive function in PD [28], while significant evidence has been gathered towards the role of cognition in gait quality, as demonstrated by dual-task gait impairments [29,30,31,32] and the impact of treatments targeting cholinergic pathways [33]. Therapeutics for cognitive and motor symptoms may aid the maintenance of HPA; for example, deep brain stimulation treatment for six months led to significant increases in walking bout lengths and variability in PwP [34]. However, such treatments often require time to ensure the optimal dosage and may not be uniformly effective across all participants. Therefore, it is important to identify other factors that facilitate HPA, such as PwP’s psychosocial wellbeing and support from their loved ones. Surprisingly, worse perceived cognition was associated with a greater number of steps per day, while worse motor disease severity was associated with increased walking bout lengths over time. This appears counter-intuitive, but it is possible that in this early stage of disease, PwP are motivated to participate in more HPA if they are worried about their cognition or physical health, due to public messaging around the benefits of physical activity [35].

Interestingly, worse perceived social support was associated with increases in alpha scores (indicating that less social support was associated with taking a higher proportion of short walking bouts compared to long), partially supporting hypothesis 2. In this instance, social support refers to PwP’s close personal relationships and their feelings of support from family, close friends and spouses or partners [22]. Social support may be important to PwP as friends and family often actively encourage participation with HPA or implicitly motivate others through their own behaviours or by engaging in social events which require HPA, such as travelling outside the home. Previous studies have shown that greater social isolation is associated with lower HPA volumes and intensities in older adults, independent of gender, age, socioeconomic or marital status, health or mobility problems, or depression and loneliness [36]. Similarly, a moderate level of social support is associated with more time spent in leisure-based PA and greater enjoyment in HPA participation [37]. Speculatively, high alpha scores may reflect greater amounts of activity in constrained settings, such as within the home and potentially less time in the community [18]. These findings, therefore, might be explained as people participating in fewer social events and relationships are likely to spend more time within the home. This finding indicates that there are associations between psychosocial characteristics and HPA in PwP. Future research should consider the efficacy of community or social-based programmes which encourage behaviours such as walking or other forms of physical activity, when aiming to maintain function and independence following PA diagnosis. 

### 4.3. Do Carers’ Psychosocial Outcomes Influence Habitual Physical Activity?

There were no significant differences between PwP with and without carers for HPA outcomes, nor was presence of a carer a significant predictor of change in HPA. However, trends indicated that PwP with carers show greater decline in HPA over an 18-month period. In agreement with hypothesis 3, carer anxiety and depression were associated with volume of HPA; interestingly, better perceived anxiety and depression at baseline were associated with greater decrease in steps and bouts taken per day after 18 months. Worse carer self-care was also associated with greater decrease in volume of HPA in PwP. This is the first study to demonstrate a relationship between carer psychosocial status and HPA in PD [3,5,6,7,8]. In PD, carers have dynamic roles, which evolve and become increasingly important throughout the disease course, providing emotional, social, and functional support to the PwP [11,12]. While carers with better psychosocial wellbeing may initially support and facilitate HPA participation, their evolving role may lead to an exacerbation of HPA decline as their caring responsibilities progress. Approximately 22% and 9% of PD carers experience significant anxiety and depression, respectively, which are associated with greater PD disease severity, cognitive and functional problems, and changes to their lifestyle and relationships with their care recipient [11,38]. This relationship may be reciprocal, with greater functional problems leading to worse anxiety and depression, and poorer carer psychosocial experiences exacerbating functional problems in PwP. Similarly, carers are often restricted in their own pursuits of leisure activity or self-care, and often prioritise the needs of the PwP to protect them from accidents or ill health; these protective mechanisms may also act as a barrier to HPA for PwP, potentially explaining these findings. 

We can see similar findings with regards to pattern and variability of HPA, with greater carer depression associated with less variability, while greater carer strain is associated with higher variability in PwP. Speculatively, lower variability may indicate that people are engaging in similar activities across the measured time period, while higher variability may reflect participation in a range of different walking activities [18]. A depressed carer may not have the emotional capacity to support PwP to engage in different HPA events, while carers who do facilitate these behaviours may experience more strain in doing so. Similarly, greater carer strain is associated with an increased walking bout length after 18 months; perhaps carers are facilitating more continuous walking periods (i.e., walks around the neighbourhood or community), with time commitments leading to greater strain across other areas of life. When considering these results in the context of other progressive neurological disorders, van Alphen, et al. [39]’s systematic review on qualitative barriers and facilitators of physical activity in dementia similarly highlights the important role of carers; carers were considered key facilitators for supporting people with dementia to maintain physical activity, but greater carer burden and psychological distress were also seen as significant barriers to physical activity. These findings highlight the importance of developing personalised interventions for both PwP and their carer to allow function to be maintained for longer. In particular, interventions which support carer self-care and mental health, such as psychoeducation regarding coping strategies and respite, may be beneficial for the dyadic unit [40,41].

### 4.4. Limitations and Future Outlooks

This is the first study to demonstrate relationships between HPA outcomes and psychosocial characteristics in people with early PD and their carers, providing novel targets for intervention development. The study was strengthened by a well-characterised longitudinal cohort and the inclusion of nuanced HPA metrics such as variability and alpha. However, this was a secondary analysis on the existing ICICLE-PD dataset, limiting psychosocial measures examined. Additionally, carer measures were only employed in a subset of participants 36 months from follow-up, limiting sample size and statistical power. However, results provide proof of concept; therefore, we recommend that further research is conducted on a larger newly-diagnosed PD sample to confirm these findings, consider other relevant metrics such as sedentary behaviour, intensity of physical activities and impact of co-morbidities, and develop future HPA interventions targeting both PwP and their carer. 

Importantly, future research should account for the influences of non-motor symptoms on HPA, such as discrete cognitive impairments, and consider the impact of different disease stages and severity on HPA outcomes. Future work should also consider monitoring HPA in carers in parallel with their care recipients, in order to examine associations between the dyad. Although our algorithm for deriving HPA outcomes is validated in healthy people, it has not been validated in PD in the real world using gold-standard methods such as video analysis. As people with PD have significant gait impairments [42,43,44] such as slow gait speed and shorter toe clearance; this may reduce the accuracy of the algorithm to identify every instance of HPA. It should also be noted that discrete gait characteristics, such as those pertaining to gait variability (e.g., step time variability) demonstrate low-moderate intra-class correlation scores between the sensor and an instrumented walkway (considered gold standard). While not directly relevant to the HPA metrics this study utilized, it is worth noting that this may also impact accuracy of our findings; this should be considered a limitation, and a validation study may be informative as research in this area moves forward. Additionally, the impact of environment in which participants are walking is currently unknown and poses a challenge in this current field of research. In the future, multi-sensor behaviour monitoring systems, such as accelerometers paired with smart-homes and outdoor-GPS monitoring, may provide a wealth of contextual information which would allow us to understand where and when people are active, and how this corresponds with carer movements [45,46]. These systems may provide data on non-motor activities in Parkinson’s disease, such as sleep behaviours and neuropsychiatric symptoms, which would allow a more holistic understanding of the impact of carers on multiple everyday behaviours [47]. We also recommend the addition of a questionnaire to assess the acceptability of wearing a small body-worn monitor continuously to capture HPA data, which would be informative as we move forward in the digital mobility field. We recommend that future research should consider collecting a comprehensive battery of psychosocial measures in both people with PD and their carers longitudinally to further explore the impact of these characteristics on HPA.

## 5. Conclusions

In PD, preliminary evidence indicates that psychosocial experiences of both PwP and their carers are associated with decline in HPA over time. This suggests that interventions to improve HPA and function should target more than the physical impairments in PwP, addressing the emotional wellbeing of the dyadic unit. Future research should consider the role of adaptive coping strategies for PwP and their carer to support the retention of cognitive, functional, and psychosocial status. 

## Figures and Tables

**Table 1:** Demographic, clinical and cognitive information of participants with Parkinson’s disease.

	Overall PD Group		PD with No Carer		PD with Carer		*p*-Value
	n = 64		n = 24		n = 40		
Age	68.9	(10.0)	64.5	(10.7)	71.5	(8.7)	**0.006**
Sex (n, %f)	23	(35.9)	13	(54.2)	10	(25.0)	**0.019**
Months since diagnosis (36-month follow-up)	44.3	(4.3)	43.9	(4.1)	44.6	(4.4)	0.488
Faller status (n, % Yes)	35	(54.7)	14	(58.3)	21	(52.5)	0.630
MDS-UPDRS-III	37.4	(11.8)	34.5	(11.9)	39.1	(11.5)	0.139
H&Y	2	(2–2)	2	(2–2)	2	(2–2)	0.465
LEDD (mg/day)	535.2	(294.3)	515.9	(360.3)	546.7	(250.9)	0.688
Step Velocity (m/s)	1.1	(1.0–1.3)	1.2	(1.0–1.4)	2.0	(1.0–1.3)	0.267
MoCA	27	(24–29)	28	(25–29.5)	26	(24–28)	0.135
FAS	36	(27–45.5)	38.5	(30.5–51)	34.5	(25.5–45)	0.172
GDS-15	2	(1–4)	25	(0–3.5)	3	(1.5–4.5)	0.135
PDQ-39 SI	21.1	(15.6)	18.1	(14.9)	34.5	(15.9)	0.227
PDQ-39 Mobility	24.9	(25.5)	19.3	(19.7)	28.3	(28.1)	0.172
PDQ-39 ADL	24.6	(20.7)	22.0	(18.4)	26.1	(22.1)	0.448
PDQ-39 Emotion	20.1	(18.6)	17.7	(20.3)	21.5	(17.6)	0.439
PDQ-39 Stigma	11.2	(13.2)	10.9	(12.9)	11.4	(13.5)	0.892
PDQ-39 Support	8.1	(14.1)	10.1	(16.7)	6.9	(12.4)	0.384
PDQ-39 Cognition	25.8	(20.3)	18.0	(17.0)	30.5	(20.9)	**0.016**
PDQ-39 Communication	16.8	(20.3)	13.5	(18.2)	18.8	(21.5)	0.325
PDQ-39 Body Discomfort	33.9	(24.1)	33.0	(21.6)	34.4	(25.7)	0.826

Figures presented are mean (SD) or median (lower quartile–upper quartile), unless otherwise stated. Significant values refer to differences between people with PD with carers and without carers. F = female, MDS-UPDRS-III = Movement Disorders Society Unified Parkinson’s disease rating scale, H&Y = Hoehn and Yahr staging, LEDD = Levodopa equivalent dose, m/s = metres per second, MoCA = Montreal Cognitive Assessment, FAS = FAS verbal fluency test, GDS-15 = Geriatric depression scale, PDQ-39 = Parkinson’s disease questionnaire 39.

**Table 2:** Habitual physical activity in people with Parkinson’s disease with and without carers at two timepoints.

		Overall PD Group (n = 64 at Baseline)		No Carer (n = 24 at Baseline)		With Carer (n = 40 at Baseline)		No Carer vs. Carer
		Median	Lower-Upper Quartile	Median	Lower-Upper Quartile	Median	Lower-Upper Quartile	*p*-Value
Real-world Step Velocity	Baseline (n = 64)	1.02	0.96–1.08	1.04	0.99–1.11	1.02	0.93–1.07	0.088
	Follow up (n = 48)	1.00	0.095–1.10	1.04	0.97–1.11	0.98	0.94–1.07	0.109
Walk Time Per day (min)	Baseline (n = 64)	161.3	120.3–212.9	180.0	110.8–221.6	149.3	120.3–201.0	0.446
	Follow-up (n = 48)	137.4	111.2–192.3	152.9	120.7–214.8	135.5	110.8–181.2	0.359
Steps Per Day	Baseline (n = 64)	10,549.8	8018.3–14,197.2	11,428.6	8306.4–16,059.1	9901.9	7856.7–13,285.2	0.292
	Follow-up (n = 48)	9907.0	7940.1–13,112.5	10,257.3	8784.3–14,470.7	9669.1	7839.6–12,372.8	0.454
Bouts Per Day	Baseline (n = 64)	608.7	470.3–720.3	630.9	477.8–746.6	603.6	466.1–720.3	0.830
	Follow-up (n = 48)	543.4	425.5–705.6	603.9	437.3–762.3	538.6	416.7–665.1	0.562
Mean Bout Length (s)	Baseline (n = 64)	15.7	14.1–18.7	16.9	14.3–20.9	15.5	14.0–17.7	0.233
	Follow-up (n = 48)	16.0	13.5–18.3	16.0	13.8–17.7	16.2	13.0–18.8	0.941
Variability	Baseline (n = 64)	0.8	0.8–0.9	0.8	0.8–1.0	0.8	0.8–0.9	0.579
	Follow-up (n = 48)	0.8	0.8–0.9	0.8	0.8–0.9	0.8	0.8–0.9	0.767
Alpha	Baseline (n = 64)	1.6	1.6–1.7	1.6	1.6–1.7	1.7	1.6–1.7	0.039
	Follow-up (n = 48)	1.6	1.6–1.7	1.6	1.6–1.7	1.6	1.6–1.7	0.245

*p*-values refer to differences between people with Parkinson’s disease with carers and without carers based on Mann–Whitney U tests. No significant results after Benjamini–Hochberg procedure.

**Table 3:** Significant baseline cognitive, physical, and psychosocial explanatory variables of change in habitual activity in the overall Parkinson’s disease group.

	β	SE	*t*-Value	*p*-Value
**Time Spent Walking Per Day (min)**				
MDS-UPDRS III	1.5	0.7	2.1	0.044
FAS	1.9	0.5	3.5	**0.001**
Step velocity (m/s)	169.9	28.7	5.9	**<0.001**
Time	7.8	13.7	0.6	0.571
PDQ-39 Cognition	1.1	0.4	2.8	**0.006**
PDQ-39 Cognition × Time	−1.0	0.4	−2.4	0.018
**Steps Per Day**				
MDS-UPDRS III	127	52.8	2.4	0.019
FAS	121	39.9	3.0	**0.003**
Step velocity (m/s)	12,522	2105.6	5.9	**<0.001**
Time	978	992.0	1.0	0.328
PDQ-39 Cognition	68.1	29.9	2.3	0.025
PDQ-39 Cognition × Time	−64	29.7	−2.2	0.034
**Bouts Per Day**				
FAS	4.6	1.5	3.0	**0.004**
Step velocity (m/s)	301.6	87.7	3.4	**0.001**
**Mean Bout Length (s)**				
MDS-UPDRS III	0.0	0.1	0.1	0.898
MDS-UPDRS III × Time	0.2	0.1	2.7	**0.009**
MoCA	0.3	0.2	1.1	0.254
MoCA × Time	−0.7	0.3	−2.3	0.025
FAS	−0.1	0.1	−1.9	0.057
FAS × Time	0.2	0.1	3.7	**0.001**
Step velocity (m/s)	5.7	3.2	1.8	0.079
Step velocity (m/s) × Time	9.5	4.2	2.2	0.029
Time	−10.2	8.8	−1.2	0.251
**Variability**				
MDS-UPDRS III	0.001	0.001	0.5	0.587
MDS-UPDRS III × Time	0.004	0.002	2.6	0.013
GDS-15	0.005	0.005	0.9	0.352
GDS-15 × Time	−0.016	0.007	−2.3	0.023
FAS	−0.002	0.001	−1.6	0.103
FAS × Time	0.002	0.001	2.2	0.031
Time	−0.191	0.079	−2.4	0.019
Step velocity (m/s)	0.149	0.045	3.3	**0.001**
**Alpha**				
Age	0.002	0.001	3.9	**<0.001**
Time	0.002	0.008	0.2	0.822
PDQ-39 Social Support	0.000	0.000	0.1	0.941
PDQ-39 Social Support × Time	0.001	0.001	2.9	**0.005**

Significant results after Benjamini–Hochberg procedure are highlighted in bold, *p* ≤ 0.01. MoCA = Montreal Cognitive Assessment, FAS = FAS verbal fluency test, PDQ-39 = Parkinson’s disease questionnaire, GDS-15 = Geriatric depression scale. Entered into models: Age × Time + FAS × Time + GDS-15 × Time+ MoCA × Time + Mean step velocity × Time + Sex + MDS-UPDRS III × Time + Carer × Time + PDQ-39 Mobility × Time + PDQ-39 ADL × Time + PDQ-39 Emotion × Time + PDQ-39 Stigma × Time + PDQ-39 Social support × Time + PDQ-39 Cognition × Time + PDQ-39 Communications × Time + PDQ-39 Bodily discomfort × Time.

**Table 4:** Significant baseline cognitive, physical and psychosocial explanatory variables of change in habitual activity in people with Parkinson’s disease with carers only.

	β	SE	*t*-Value	*p*-Value
**Time Spent Walking Per Day (min)**				
Step velocity (m/s)	138.3	30.7	4.5	**<0.001**
Time	−16.0	11.6	−1.4	0.180
Hours caregiving per week	0.4	0.1	2.7	0.011
Hours caregiving per week × Time	−0.4	0.1	−2.6	0.017
PDQ-C Anxiety and Depression	1.0	0.6	1.8	0.089
HADS-A	−5.4	1.9	−2.8	**0.008**
**Steps Per Day**				
Step velocity (m/s)	10,949.5	2342.2	4.7	**<0.001**
Time	−1389.0	790.7	−1.8	0.092
Hours caregiving per week	−19.9	13.0	−1.5	0.136
Hours caregiving per week × Time	23.8	11.1	2.1	0.042
PDQ-C Anxiety and Depression	−69.0	70.3	−1.0	0.333
PDQ-C Anxiety and Depression × Time	222.4	72.6	3.1	**0.005**
PDQ-C Self care	149.6	57.5	2.6	0.013
PDQ-C Self-care × Time	−240.8	58.8	−4.1	**<0.001**
**Bouts Per Day**				
Step velocity (m/s)	415.2	99.4	4.2	**<0.001**
FAS	4.3	1.5	2.8	**0.009**
Time	−141.5	53.4	−2.6	0.014
Hours caregiving per week	−0.5	0.7	−0.8	0.424
Hours caregiving per week × Time	1.6	0.8	2.1	0.051
HADS-A	−18.2	6.9	−2.7	0.012
PDQ-C Anxiety and Depression	3.0	3.8	0.8	0.437
PDQ-C Anxiety and Depression × Time	14.0	4.8	2.9	**0.007**
PDQ-C Self care	2.8	2.9	1.0	0.343
PDQ-C Self-care × Time	−13.3	4.0	−3.4	**0.002**
**Mean Bout Length (s)**				
Step velocity (m/s)	4.8	2.2	2.1	0.039
Step velocity (m/s) × Time	7.2	2.4	3.0	**0.006**
FAS	0.0	0.0	−0.9	0.372
FAS × Time	0.1	0.0	3.4	**0.003**
MoCA	0.2	0.2	1.3	0.194
MoCA × Time	−1.0	0.2	−5.2	**<0.001**
Carer Age (years)	0.0	0.1	−0.9	0.398
Carer Age (years) × Time	−0.1	0.1	−2.7	0.012
Time	21.7	4.8	4.5	**<0.001**
Hours caregiving per week	0.1	0.0	3.5	**0.001**
Hours caregiving per week × Time	−0.1	0.0	−5.8	**<0.001**
PDQ-C Strain	0.0	0.0	−1.4	0.164
PDQ-C Strain × Time	0.2	0.0	5.0	**0.000**
**Variability**				
Carer Age (years)	0.000	0.001	0.3	0.789
Carer Age (years) × Time	−0.003	0.001	−2.2	0.038
Time	0.192	0.088	2.2	0.038
HADS-D	−0.010	0.004	−2.8	**0.009**
PDQ-C Strain	0.001	0.000	3.0	**0.005**
**Alpha**				
Time	0.003	0.008	0.4	0.672
PDQ-39 Social support	0.000	0.001	−0.2	0.844
PDQ-39 Social support × Time	0.002	0.001	2.8	**0.010**
HADS-D	0.005	0.002	2.4	0.022
PDQ-C Strain	−0.001	0.000	−1.8	0.083

Significant results after Benjamini–Hochberg procedure are highlighted in bold, *p* ≤ 0.01. MoCA = Montreal Cognitive Assessment, PDQ-39 = Parkinson’s disease questionnaire, PDQ-C = Parkinson’s disease questionnaire for carers, HADS = Hospital Anxiety and Depression Scale, HADS-A = HADS anxiety subscale, HADS-D = HADS depression subscale, FAS = FAS verbal fluency test.

## Data Availability

The data presented in this study are available on request from the corresponding author. The data are not publicly available due to ongoing analysis.

## Supplementary Materials

The following supporting information can be downloaded at https://www.mdpi.com/article/10.3390/s22030871/s1, Table S1: Table of abbreviations. Table S2: Demographic information about carers partaking in the study.
