# Supplementary material for: Factors Influencing Habitual Physical Activity in Parkinson’s Disease: Considering the Psychosocial State and Wellbeing of People with Parkinson’s and Their Carers
Source: Sensors (Basel). 2022 Jan 24;22(3):871. doi: 10.3390/s22030871 (PMC8837970; doi:10.3390/s22030871)
Supplement: Supplementary file 1 [file sensors-22-00871-s001.zip › sensors-1521730-supplementary.pdf]

Table S1. Table of abbreviations.

Abbreviation	Definition
HPA	Habitual Physical Activity
PD	Parkinson's disease
QoL	Quality of Life
PwP	People with Parkinson's disease
ICICLE-PD	Incidence of Cognitive Impairment in Cohorts of Longitudinal Evaluation–Parkinson's disease study
MDS-UPDRS III	Movement Disorders Society Unified Parkinson's Disease Rating Scale Part III
GDS-15	Geriatric Depression Scale
LEDD	Levodopa Equivalent Dose
PDQ	Parkinson's disease questionnaire
MoCA	Montreal Cognitive Assessment
FAS	FAS Verbal Fluency Test
HADS	Hospital Anxiety and Depression Scale
NPI	Neuropsychiatric Inventory
SQLC	Scale of Quality of Life of Caregivers

Table S2. Demographic information about caregivers partaking in the study.

	Caregiver
Age (years)	67±10
Sex (%f)	73%
Length of time as caregiver (months)	24±26
Time spent caregiving (hours/week)	40±63
OARS (number of health problems)	2±1
HADS Anxiety (0-21)	4±4
HADS depression (0-21)	3±3
SQLC (0-149)	116±13
PDQ SI	17.0±17.0
PDQ Personal and Social	16.1±15.1
PDQ Anxiety and Depression	16.7±15.5
PDQ Self-care	18.2±24.1
PDQ Strain	24.0±27.6
NPI Distress (0-60)	4±5

Figures are mean±SD unless otherwise stated. F = female, OARS = Older American Resources and Services Physical Health Checklist, HADS = Hospital Anxiety and Depression Scale, SQLC = Scale of QoL of Caregivers, PDQ = Parkinson's disease questionnaire carer version, NPI Distress = Neuropsychiatric inventory carer distress scale.
